# Adherence to physical activity in adults with chronic diseases: *ELSA-Brasil*

**DOI:** 10.11606/S1518-8787.2018052000215

**Published:** 2018-03-14

**Authors:** Ludimila Forechi, José Geraldo Mill, Rosane Härter Griep, Itamar Santos, Francisco Pitanga, Maria del Carmen Bisi Molina

**Affiliations:** I Universidade Federal de Juiz de Fora. Departamento de Fisioterapia. Governador Valadares, MG, Brasil; II Universidade Federal do Espírito Santo. Departamento de Ciências Fisiológicas. Programa de Pós-Graduação em Saúde Coletiva. Vitória, ES, Brasil; III Fundação Oswaldo Cruz. Instituto Oswaldo Cruz. Laboratório de Educação em Ambiente e Saúde. Rio de Janeiro, RJ, Brasil; IV Universidade de São Paulo. Faculdade de Medicina. Departamento de Clínica Médica. São Paulo, SP, Brasil; V Universidade Federal da Bahia. Faculdade de Educação. Departamento de Educação Física. Salvador, BA, Brasil; VI Universidade Federal do Espírito Santo. Departamento de Educação Integrada em Saúde. Programa de Pós-Graduação em Saúde Coletiva. Vitória, ES, Brasil

**Keywords:** Exercise, Patient Compliance, Patient Dropouts, Risk Factors, Socioeconomic, Factors Dyslipidemias, prevention & control, Diabetes Mellitus, prevention & control

## Abstract

**OBJECTIVE:**

The objective of this study is to investigate the adherence and the factors that influence adherence to physical activity in adults with dyslipidemia, hypertension, or diabetes.

**METHODS:**

The analyses were based on data collected at the baseline of the 14,521 participants from the study *ELSA-Brasil* aged between 35 and 74 years. The level of leisure time physical activity was determined using the International Physical Activity Questionnaire. Logistic regression analyses were performed to examine the influence of the demographic data, socioeconomic conditions, perceived health status, and access to exercise facilities in the neighborhood on adherence to physical activity.

**RESULTS:**

Men with hypertension and dyslipidemia were more active than women. The results show that 17.8%, 15.1%, and 13.9% of the subjects who reported dyslipidemia, hypertension, and diabetes, respectively, adhere to the physical activity recommendations. The factors positively associated with adherence were higher education and income. Older individuals who reported poor perceived health, were overweight and obese, regularly smoked, and had fewer opportunities to exercise in the neighborhood presented lower adherence.

**CONCLUSIONS:**

The number of adults with dyslipidemia, hypertension, and diabetes who adhere to the physical activity recommendations is very low. Higher education and income are positively associated with adherence, while age, excess body weight, negative perceived health, regular smoking, and lack of opportunity to exercise in the neighborhood were considered barriers to physical activity.

## INTRODUCTION

Noncommunicable chronic diseases (NCD) are the leading cause of morbidity and mortality worldwide^1^. Many studies have shown that regular aerobic exercises can prevent or change the natural history of several NCD, including hypertension, diabetes, and dyslipidemia. However, the number of individuals who regularly attend aerobic exercise programs is relatively low. This low adherence to regular exercise may be attributed to several factors. A better understanding of these factors is important for the healthcare system because NCD are highly prevalent in adults and because the adoption of adequate lifestyle habits, such as regular exercise, may reduce the incidence of these diseases, as well as health costs. The main fifteen global risk factors explain half the global mortality and more than a third of the global disability-adjusted life years (DALY); high levels of blood pressure, plasma glucose, and cholesterol represent the second, third, and seventh leading risk factors, respectively, in the Latin America and Caribbean countries^2^. Quantifying the relationships of these risk factors with overall morbidity and mortality is important because most cardiovascular outcomes associated with these factors, e.g., myocardial infarction and stroke, may be prevented with long-term adherence to physical activity (PA) programs^3^.

For adults, the current recommendations regarding PA are at least 150 minutes of moderate-intensity aerobic PA per week, at least 75 minutes of vigorous-intensity aerobic PA per week, or an equivalent combination of the two. Aerobic activities should be performed in at least 10-minute intervals and can include leisure time physical activity (LTPA), transportation, occupational work, and housework^4^.

The benefits of regular PA for the prevention and treatment of hypertension, diabetes, and dyslipidemia are well documented. Regular PA in patients with type 2 diabetes reduces the risk of stroke, renal failure, and peripheral thrombotic events^5^. Aerobic exercise also improves glucose control in patients with type 2 diabetes^6^. In hypertensive patients, PA has been associated with better blood pressure control and the regression or prevention of left ventricular hypertrophy and arterial stiffening. Epidemiological and experimental data also support the favorable effect of regular exercise on lipoprotein profiles and thus the prevention of atherosclerosis.

Despite these benefits, recent estimates suggest that only 31.1% of adults adhere to the actual PA recommendations^8^. Furthermore, although older adults have a higher prevalence of NCD and would benefit more from PA, physical inactivity is more common in this population, and adherence to PA recommendations may thus be even lower in older groups^9^. However, studies on the factors that influence adherence to PA recommendations are scarce, especially in the Brazilian population. Adherence is affected by a complex interaction of social, emotional, environmental, and psychological factors^10^, and understanding these factors is of great importance for healthcare professionals. Therefore, in this study, we sought to determine the rates of adherence to the recommended levels of LTPA and the factors that influence this adherence in participants with self-reported hypertension, diabetes, or dyslipidemia at the baseline of the Brazilian Longitudinal Study of Adult Health (*ELSA-Brasil*).

## METHODS

### Study Population

The *ELSA-Brasil* was designed to investigate the incidence and determinants of chronic diseases in the Brazilian population using a prospective cohort^11^. This cohort consisted of 15,105 civil servants (35–74 years old, active or retired) from six public teaching and research institutions in six Brazilian cities. Baseline data were collected from 2008 to 2010. The project was approved by all of the ethic committees of the institutions from which the data were collected. All participants signed the informed consent before responding to the questionnaires, which were completed during a face-to-face interview, and undergoing the clinical and laboratory exams. The main characteristics of the cohort at baseline have been published elsewhere^12^. Previous diagnoses of hypertension, diabetes, and dyslipidemia were obtained by self-report during the interview.

### PA Assessment

Physical activity was determined by the LTPA domain of the International Physical Activity Questionnaire (IPAQ). This instrument has been validated in the Brazilian population and includes questions regarding the frequency, duration, and intensity of activities lasting ten or more minutes^13^. The IPAQ includes activities performed during leisure time and for transportation. Moderate-intensity PA refers to tasks that require moderate physical effort (a small increase in breath rate compared with that at rest). Vigorous-intensity PA refers to tasks that require substantial physical and respiratory efforts. Moderate- and vigorous-intensity PA were reported in min/week.

Adherence to PA guidelines^4^ was defined as total PA time ≥ 150 min/week [(minutes of moderate-intensity PA/week) + (minutes of vigorous-intensity PA/week) × 2]. Participants with lower values were considered non-adherent to PA recommendations.

### Covariates

We tested the influence of four groups of covariates that may affect adherence to PA: demographic characteristics, socioeconomic condition, perceived health status, and access to exercise facilities in the participant’s neighborhood of residence.

The demographic characteristics included age, marital status, and self-declared race. Age was categorized into decades (35–44, 45–54, 55–64, or 65–74 years old), marital status was categorized into married/living with partner, divorced/widowed, or single, and self-declared race was categorized into white or non-white.

Socioeconomic condition included years of education (less than high school, high school, or college), employment situation (active or retired), occupation (unskilled, technical/clerical, or faculty and professional staff), and monthly family income (in US$: < 505, 505 to 1,250, or > 1,250). The income of the participants was obtained in Brazilian Reais (BRL), which were converted to US$ at a rate of BRL 2 = US$1.

The covariates of health status included self-perceived health (reported as very good or good, fair, or poor or very poor), smoking status (never, former, or regular), alcohol use (never, former, or regular), morbidity score (presence of diabetes, hypertension, or dyslipidemia – 1, 2, or 3 of these conditions), presence of joint disease (yes or no), and body mass index (BMI), defined as body weight divided by height squared (kg/m^2^). Body weight was measured with an electronic scale (Toledo, Brazil), and height was assessed with a wall-mounted stadiometer (Seca, Germany). The BMI was classified into three categories according to the WHO guidelines: underweight/normal (≤ 25 kg/m^2^), overweight (25–29.9 kg/m^2^), and obese (≥ 30 kg/m^2^)^12^.

Three factors were investigated regarding the participant’s neighborhood: opportunity to practice PA, seeing persons practicing PA, and facilities to practice PA^14^. These factors were evaluated by three consecutive statements: “My neighborhood offers many opportunities to be physically active”, “I often see other people exercising in my neighborhood”, and “Local sports clubs and other facilities in my neighborhood offer many opportunities to get exercise”. The participant was instructed to choose the best answer among the following: 1 – strongly agree, 2 – partially agree, 3 – neither agree nor disagree, 4 – partially disagree, and 5 –strongly disagree. For this study, responses 1 and 2 were merged into “agree” and 4 and 5 were merged into “disagree”.

### Statistical Analysis

Categorical variables were described as absolute counts and proportions. Chi-square tests were used for the bivariate analysis of the associations between the characteristics of participants and adherence to PA recommendations.

Logistic regression analyses were performed to estimate the crude and adjusted odds ratios (OR) and 95% confidence intervals (95%CI) of physical activity in men and women. The variables included in the logistic regression models were selected based on the statistical criterion of p < 0.05 in the chi-square test. Logistic regression without adjustment (crude OR) was performed to evaluate the association of demographic characteristics, socioeconomic condition, perceived health status, and access to exercise facilities in the neighborhood with adherence to PA recommendations among subjects with self-reported dyslipidemia, hypertension, or diabetes. Considering the presence of possible confounders, six different adjusted logistic regression models were tested. In these analyses, we selected the variables that did not include zero in their 95%CI. All analyses were stratified by gender and performed in SPSS 18.0 (Chicago, IL, USA). The significance level was set at p < 0.05.

## RESULTS

This analysis was limited to the 14,521 participants (96.1% of the cohort) with complete data. We excluded subjects with incomplete IPAQ (n = 223), those with no information regarding a previous diagnosis of hypertension (n = 4), diabetes (n = 10), or dyslipidemia (n = 31), and women who reported diabetes or hypertension only during pregnancy (n = 316).

Dyslipidemia was reported by 36.0% of the participants, hypertension by 34.7%, and diabetes by 8.9%. A total of 36.3% of participants reported only one of these diagnoses, 16.4% reported two, and 3.5% reported three. Most individuals (81%) either did not practice any type of PA or practiced some PA without reaching the WHO-recommended levels. Only 19.0% of the cohort adhered to the PA recommendations, and there were minor differences among those who reported having high cholesterol (17.8%), hypertension (15.1%), or diabetes (13.9%). Men were more likely to be active than women only among individuals who self-reported dyslipidemia or hypertension (Figure).

**Figure:**
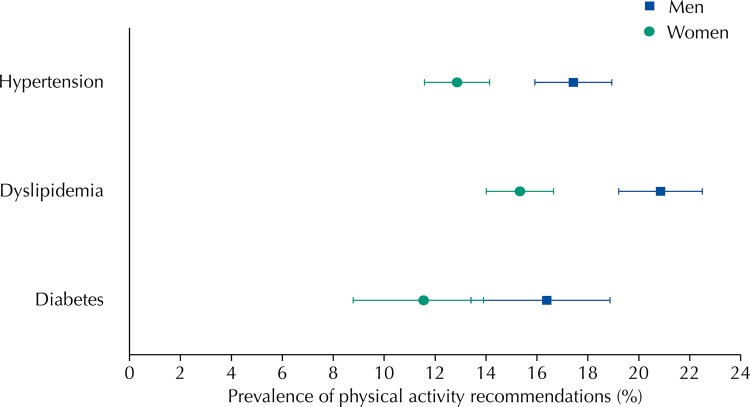
Prevalence of adherence to physical activity recommendations by gender.

### Characteristics of the Sample According to Adherence to PA

The effects of the demographic characteristics, socioeconomic conditions, health status, and access on adherence to PA are shown in Table 1. Demographic variables (age, marital status, and race [white *versus* non-white]) showed little or no influence in general, except among younger men with dyslipidemia or hypertension, who were more likely to be adherent than their older counterparts. Two socioeconomic variables (education level and income) were closely related to adherence to PA, while the other two (occupation level and employment) were unrelated to adherence. Higher education and higher income were associated with higher adherence in both men and women. Self-perceived health showed a direct relationship with adherence for all three access conditions in both genders; that is, those who reported having a lower health status were less likely to adhere to PA recommendations. Participants who complained of joint diseases, as well as those with higher body fat, were also less likely to be adherent. An interesting finding was observed in relation to smoking and alcohol intake. Specifically, adherence to PA was less likely among regular smokers, except for those who reported diabetes, whereas regular use of alcohol was associated with adherence to PA in all groups, except for women who reported diabetes. Finally, subjects with more than one risk factor were less likely to adhere to PA recommendations, regardless of gender.

**Table 1:** Demographic characteristics, socioeconomic condition, perceived health status, and access to exercise among subjects with dyslipidemia, hypertension, and diabetes meeting the physical activity (PA) recommendations in the ELSA-Brasil cohort.

Variable	Dyslipidemia (n = 5,232)				Hypertension (n = 5,040)				Diabetes (n = 1,291)			
		
	Men (n = 2,369)		Women (n = 2,863)		Men (n = 2,427)		Women (n = 2,613)		Men (n = 700)		Women (n = 591)	
					
	n (%)	p	n (%)	p	n (%)	p	n (%)	p	n (%)	p	n (%)	p
Demographic characteristics												

Age group (years)		< 0.001		0.182		< 0.001		0.595		0.474		0.662
35–44	98 (27.3)		46 (13.9)		61 (23.6)		25 (10.2)		8 (16.3)		2 (6.9)	
45–54	200 (21.6)		154 (15.5)		156 (18.4)		117 (13.2)		36 (16.8)		22 (12.9)	
55–64	128 (17.0)		184 (16.7)		112 (13.0)		137 (13.3)		40 (13.8)		25 (10.1)	
65–74	68 (20.5)		55 (12.5)		94 (20.5)		57 (12.6)		29 (19.6)		18 (12.5)	
Marital status		0.521		0.812		0.409		0.285		0.605		0.075
Married/Union	408 (20.8)		211 (15.2)		361 (17.8)		172 (14.0)		94 (16.4)		33 (12.0)	
Divorced	66 (22.6)		155 (15.0)		42 (14.6)		119 (11.9)		13 (13.4)		19 (8.3)	
Single	20 (17.5)		73 (16.3)		20 (18.3)		45 (11.8)		6 (20.7)		15 (17.2)	
Race		0.010		0.277		0.435		0.947		0.496		0.357
White	271 (23.0)		222 (14.7)		214 (18.0)		180 (12.8)		59 (17.3)		33 (10.2)	
Non-white	217 (18.7)		212 (16.2)		(16.8)		150 (12.7)		54 (15.3)		33 (12.6)	

Socioeconomic condition												

Years of education		< 0.001		< 0.001		< 0.001		< 0.001		< 0.001		0.010
Less than high school	19 (9.6)		5 (3.3)		22 (8.4)		8 (4.2)		5 (4.4)		1 (1.7)	
High school	24 (12.2)		13 (7.1)		22 (9.3)		22 (10.2)		10 (13.3)		4 (6.0)	
College	451 (22.8)		421 (16.7)		379 (19.7)		306 (13.9)		98 (19.2)		62 (13.3)	
Employment situation		0.439		0.420		0.334		0.109		0.060		0.427
Active	397 (21.2)		284 (14.9)		306 (17.0)		205 (12.1)		69 (14.4)		33 (10.4)	
Retired	97 (19.6)		155 (16.1)		117 (18.7)		131 (14.3)		44 (20.0)		34 (12.5)	
Occupation		0.149		0.904		0.358		0.193		0.350		0.618
Unskilled	127 (20.0)		132 (15.4)		111 (17.0)		100 (13.8)		34 (19.0)		17 (10.9)	
Technical/Clerical	171 (19.4)		147 (14.9)		151 (16.4)		107 (11.3)		38 (13.9)		28 (13.0)	
Faculty/Professional staff	196 (23.0)		160 (15.7)		161 (18.9)		129 (13.7)		41 (16.5)		22 (10.0)	
Per capita income (USD)		< 0.001		< 0.001		< 0.001		< 0.001		< 0.001		< 0.001
< 505.00	123 (14.5)		76 (8.1)		134 (13.6)		90 (8.6)		30 (9.6)		12 (5.1)	
505.00–1,250.00	200 (21.5)		178 (15.4)		159 (17.8)		120 (12.0)		39 (17.0)		32 (13.4)	
> 1,250.00	170 (29.1)		182 (24.5)		129 (23.7)		126 (22.9)		43 (28.9)		23 (20.0)	

Health status and health-related behaviors												

Self-perceived health		< 0.001		< 0.001		< 0.001		< 0.001		0.002		0.002
Very good/Good	417 (23.7)		371 (17.8)		344 (21.0)		267 (15.1)		80 (20.3)		45 (16.0)	
Fair	70 (12.8)		63 (9.4)		69 (9.7)		68 (9.3)		26 (10.0)		20 (7.8)	
Poor/Very poor	6 (9.7)		5 (4.5)		9 (12.5)		1 (0.9)		6 (14.0)		2 (3.8)	
Smoking status		< 0.001		0.046		0.010		0.060		0.375		0.305
Never	259 (24.2)		273 (15.8)		204 (20.0)		211 (13.2)		48 (18.7)		46 (13.0)	
Former	188 (19.1)		129 (16.2)		175 (16.3)		100 (13.9)		51 (15.0)		15 (8.6)	
Regular	47 (14.8)		37 (10.8)		44 (13.4)		25 (8.6)		14 (13.9)		6 (9.7)	
Alcohol use		0.005		< 0.001		< 0.001		< 0.001		0.005		0.230
Never	12 (13.0)		55 (11.4)		11 (10.6)		52 (10.4)		7 (17.5)		21 (14.3)	
Former	68 (16.3)		81 (12.5)		68 (13.0)		61 (9.3)		20 (9.3)		15 (8.3)	
Regular	413 (22.2)		303 (17.6)		343 (19.1)		223 (15.4)		85 (19.2)		31 (11.8)	
Morbidity score		< 0.001		< 0.001		0.125		0.017		0.245		0.053
1	286 (24.3)		265 (17.2)		193 (17.4)		152 (12.5)		25 (16.9)		11 (12.5)	
2	174 (18.6)		154 (14.4)		196 (18.5)		164 (14.3)		54 (18.4)		36 (14.6)	
3	34 (13.2)		20 (7.8)		34 (13.2)		20 (7.8)		34 (13.2)		20 (7.8)	
Joint diseases		0.117		0.003		0.948		0.008		0.821		0.239
No	396 (20.3)		312 (16.8)		347 (17.5)		236 (14.2)		93 (16.3)		45 (12.6)	
Yes	97 (23.7)		127 (12.7)		76 (17.4)		100 (10.6)		20 (15.5)		22 (9.5)	
Body mass index		0.006		< 0.001		0.036		< 0.001		0.446		0.401
Underweight/Normal	165 (24.9)		195 (20.9)		105 (19.7)		116 (19.8)		25 (17.9)		15 (15.2)	
Overweight	224 (20.0)		165 (14.7)		204 (18.3)		126 (12.4)		58 (17.1)		23 (11.1)	
Obese	105 (17.9)		79 (9.8)		114 (14.7)		94 (9.3)		30 (13.6)		29 (10.2)	

Access to exercise in neighborhood												

Opportunity to practice PA		0.018		< 0.001		< 0.001		< 0.001		0.013		0.120
Agree	388 (22.2)		365 (17.4)		340 (19.6)		268 (14.7)		92 (18.8)		51 (13.3)	
Disagree	97 (17.4)		69 (10.0)		74 (12.2)		61 (8.6)		19 (10.6)		14 (7.7)	
Neither agree nor disagree	9 (13.8)		5 (7.6)		9 (11.1)		7 (8.9)		2 (6.5)		2 (7.7)	
See persons practicing PA		0.725		0.001		0.603		0.006		0.143		0.017
Agree	403 (21.0)		360 (16.5)		345 (17.8)		278 (14.0)		99 (17.4)		53 (11.9)	
Disagree	72 (19.5)		65 (10.6)		63 (15.7)		51 (9.1)		11 (9.9)		9 (6.9)	
Neither agree nor disagree	18 (22.8)		14 (19.2)		14 (16.9)		7 (10.0)		3 (15.0)		5 (29.4)	
Conditions to practice PA		0.187		< 0.001		0.029		0.004		0.449		0.290
Agree	366 (21.9)		325 (17.3)		314 (18.8)		244 (14.4)		83 (17.3)		46 (13.0)	
Disagree	104 (18.6)		90 (11.0)		92 (14.5)		74 (9.6)		25 (14.2)		18 (9.1)	
Neither agree nor disagree	24 (17.8)		24 (14.7)		17 (14.0)		18 (12.3)		5 (11.6)		3 (7.7)	

Our results clearly showed a correlation between the availability of facilities in the neighborhood and adherence to PA. Adherence was higher in those who agreed that neighborhood facilities for sports were important and in women who frequently observed persons exercising (Table 1).

### Factors Related to Adherence to PA Recommendations

Tables 2 and 3 show the OR and 95%CI for the associations of demographic characteristics, socioeconomic condition, perceived health status, and health perceptions with adherence to PA recommendations by gender.

**Table 2:** Odds ratios and 95% confidence intervals for demographic and socioeconomic factors, health status, lifestyle, and health-related behaviors associated with adherence to physical activity recommendations in men.

Variable	Crude and adjusted odds ratios in men											

	Self-reported Dyslipidemia (n = 2,369)				Self-reported Hypertension (n = 2,427)				Self-reported Diabetes (n = 700)			
		
	C OR	95%CI	A OR	95%CI	C OR	95%CI	A OR	95%CI	C OR	95%CI	A OR	95%CI
Demographic characteristics												

Age (years)												
35–44	1		1		1		1					
45–54	0.73	0.55–0.97	0.88	0.65–1.19	0.73	0.52–1.02	0.74	0.52–1.05	-	-	-	-
55–64	0.55	0.40–0.74	0.61	0.44–0.85	0.49	0.34–0.69	0.45	0.31–0.66				
65–74	0.69	0.48–0.98	0.65	0.44–0.98	0.84	0.58–1.21	0.68	0.45–1.01				
Race												
White	1		1									
Non-white	0.77	0.63–0.94	0.77	0.63–0.95	-	-	-	-	-	-	-	-

Socioeconomic conditions												

Years of education												
Less than high school	1		1		1		1		1		1	
High school	1.31	0.69–2.49	1.15	0.60–2.20	1.12	0.60–2.07	0.94	0.50–1.77	3.35	1.10–10.24	2.86	0.92–8.87
College	2.79	1.72–4.53	1.45	0.86–2.43	2.67	1.70–4.19	1.56	0.96–2.53	5.17	2.05–13.02	2.66	1.00–7.02
Per capita income (USD)												
< 505.00	1		1		1		1		1		1	
505.00–1,250.00	1.61	1.26–2.06	1.32	1.01–1.72	1.37	1.07–1.76	1.04	0.79–1.37	1.93	1.16–3.22	1.50	0.87–2.58
>1,250.00	2.41	1.85–3.13	1.92	1.43–2.60	1.96	1.50–2.57	1.39	1.01–1.91	3.84	2.29–6.44	2.57	1.45–4.55

Health status and health-related behaviors												

Self-perceived health												
Very good/Good	1		1		1		1		1		1	
Fair	0.47	0.36–0.62	0.60	0.44–0.80	0.40	0.31–0.53	0.50	0.37–0.66	0.44	0.27–0.70	0.55	0.33–0.91
Poor/Very poor	0.34	0.15–0.80	0.50	0.21–1.20	0.54	0.26–1.09	0.71	0.34–1.45	0.64	0.26–1.56	0.87	0.34–2.18
Smoking status												
Never	1		1		1		1					
Former	0.74	0.60–0.91	0.87	0.69–1.10	0.78	0.62–0.97	0.89	0.70–1.12	-	-	-	-
Regular	0.54	0.39–0.76	0.60	0.42–0.85	0.62	0.44–0.88	0.69	0.48–0.99				
Alcohol use												
Never	1		1		1		1		1			
Former	1.30	0.67–2.51	1.27	0.64–2.50	1.27	0.64–2.49	1.34	0.67–2.68	0.48	0.19–1.23	-	-
Regular	1.90	1.03–3.53	1.64	0.87–3.10	1.99	1.05–3.76	1.73	0.90–3.34	1.12	0.48–2.62		
Morbidity score												
1	1		1									
2	0.71	0.58–0.88	0.91	0.72–1.14	-	-	-	-	-	-	-	-
3	0.47	0.32–0.69	0.84	0.54–1.29								
Body mass index												
Underweight/Normal	1		1		1		1					
Overweight	0.75	0.60–0.95	0.75	0.59–0.95	0.91	0.70–1.19	0.87	0.66–1.14	-	-	-	-
Obese	0.66	0.50–0.87	0.71	0.53–0.96	0.70	0.52–0.94	0.68	0.50–0.92				

Access to exercise in neighborhood												

Opportunity to practice physical activity												
Agree	1		1		1		1		1		1	
Disagree	0.73	0.57–0.94	0.84	0.65–1.09	0.57	0.44–0.75	0.70	0.52–0.94	0.51	0.30–0.87	0.60	0.34–1.04
Neither agree nor disagree	0.56	0.27–1.15	0.82	0.39–1.71	0.51	0.25–1.04	0.69	0.33–1.43	0.30	0.07–1.27	0.39	0.09–1.70
Conditions to practice physical activity												
Agree					1		1					
Disagree					0.73	0.57–0.94	0.89	0.68–1.18				
Neither agree nor disagree	-	-	-	-	0.70	0.42–1.19	0.78	0.45–1.34	-	-	-	-

C OR: Crude OR indicates univariate logistic regression without adjustment for variables; A OR: Adjusted OR was obtained from multivariate analyses adjusted for all significant factors in the crude analysis

**Table 3:** Odds ratios and 95% confidence intervals for demographic and socioeconomic factors, health status, lifestyle, and health-related behaviors associated with adherence to physical activity recommendations in women.

Variable	Crude and adjusted odds ratios in women											

	Self-reported dyslipidemia (n = 2,863)				Self-reported hypertension (n = 2,613)				Self-reported diabetes (n = 591)			
		
	C OR	95%CI	A OR	95%CI	C OR	95%CI	A OR	95%CI	C OR	95%CI	A OR	95%CI
Socioeconomic condition												

Years of education												
Less than high school	1		1		1		1		1		1	
High school	2.26	0.79–6.50	2.01	0.69–5.84	2.58	1.12–5.94	2.32	0.99–5.41	3.68	0.40–33.91	3.55	0.37–33.60
College	5.92	2.41–14.51	3.02	1.21–7.56	3.66	1.79–7.51	2.14	1.02–4.48	8.92	1.21–65.59	5.72	0.75–43.63
*Per capita* income (USD)												
< 505.00	1		1		1		1		1		1	
505.00–1,250.00	2.06	1.55–2.74	1.53	1.14–2.06	1.46	1.09–1.95	1.18	0.87–1.59	2.89	1.45–5.75	2.17	1.06–4.43
> 1,250.00	3.69	2.77–4.92	2.49	1.82–3.39	3.18	2.37–4.26	2.27	1.65–3.13	4.67	2.23–9.77	3.03	1.39–6.58

Health status and health-related behaviors												

Self-perceived health												
Very good/Good	1		1		1		1		1		1	
Fair	0.48	0.36–0.64	0.73	0.54–0.99	0.58	0.44–0.77	0.79	0.59–1.07	0.44	0.25–0.78	0.59	0.33–1.06
Poor/Very poor	0.22	0.09–0.54	0.42	0.16–1.06	0.05	0.01–0.36	0.08	0.01–0.55	0.21	0.05–0.87	0.29	0.07–1.27
Smoking status												
Never	1		1									
Former	1.02	0.81–1.29	1.05	0.82–1.33	-	-	-	-	-	-	-	-
Regular	0.64	0.45–0.93	0.65	0.45–0.95								
Alcohol use												
Never	1				1		1					
Former	1.11	0.77–1.60	0.97	0.66–1.42	0.88	0.59–1.29	0.81	0.54–1.21	-	-	-	-
Regular	1.65	1.21–2.24	1.15	0.83–1.59	1.56	1.14–2.16	1.18	0.84–1.66				
Morbidity score												
1	1		1		1		1					
2	0.81	0.65–1.00	1.15	0.91–1.45	1.17	0.92–1.48	1.29	1.01–1.65	-	-	-	-
3	0.41	0.25–0.65	0.83	0.50–1.39	0.59	0.36–0.96	0.96	0.57–1.60				
Articular disease												
No	1		1		1		1		-	-	-	-
Yes	0.72	0.57–0.90	0.93	0.73–1.17	0.71	0.56–0.92	0.87	0.67–1.13				
Body mass index												
Underweight/Normal	1		1		1		1		-	-	-	-
Overweight	0.65	0.52–0.82	0.71	0.56–0.90	0.58	0.44–0.76	0.58	0.44–0.78				
Obese	0.41	0.31–0.55	0.53	0.39–0.71	0.41	0.31–0.56	0.50	0.36–0.67				

Access to exercise in neighborhood												

Opportunity to practice physical activity												
Agree	1		1		1		1					
Disagree	0.53	0.40–0.69	0.88	0.63–1.19	0.54	0.41–0.73	0.83	0.59–1.16	-	-	-	-
Neither agree nor disagree	0.39	0.16–0.98	0.57	0.22–1.48	0.56	0.26–1.24	0.78	0.34–1.77				
See persons practicing physical activity												
Agree	1		1		1		1		1		1	
Disagree	0.60	0.45–0.79	0.81	0.58–1.12	0.61	0.44–0.83	0.80	0.56–1.15	0.55	0.26–1.14	0.57	0.27–1.21
Neither agree nor disagree	1.20	0.66–2.17	1.42	0.74–2.71	0.68	0.31–1.50	0.65	0.29–1.49	3.07	1.04–9.07	2.87	0.91–8.99
Conditions to practice physical activity												
Agree	1		1		1		1					
Disagree	0.59	0.46–0.76	0.74	0.55–0.99	0.63	0.48–0.83	0.81	0.59–1.10	-	-	-	-
Neither agree nor disagree	0.83	0.53–1.30	1.00	0.62–1.63	0.84	0.50–1.39	1.05	0.61–1.80				

C OR: Crude OR indicates univariate logistic regression without adjustment for variables; A OR: Adjusted OR was obtained from multivariate analyses adjusted for all significant factors in the crude analysis

Both men and women with high cholesterol were less likely to meet the recommendations if they were overweight or obese, were current smokers, had a fair or poor/very poor self-perceived health status, and did not have the opportunity to practice PA in their neighborhood. In contrast, regular use of alcohol, high education level, and high per capita income showed positive associations with adherence to PA. Among men with dyslipidemia, those who were older than 44 years and who were non-white also showed decreased adherence to PA. Additionally, women who had joint diseases, did not have facilities to practice PA, and did not see persons practicing PA were less likely to be adherent. After adjusting the models for all positive and negative factors that interfered with adherence to PA in men and women with dyslipidemia, we observed a significant positive association in both genders for only high income and negative associations for fair self-perceived health, regular smoking status, and overweight or obesity. The positive association between adherence to PA and university education was confirmed only for women. Non-white males with dyslipidemia were 0.77 (95%CI 0.63–0.95) times less likely to be adherent than their white counterparts. As expected, age was inversely related to adherence to PA in men.

In men with hypertension, an age of 55–64 years (OR = 0.49, 95%CI 0.34–0.69), fair self-perceived health (OR = 0.40, 95%CI 0.31–0.53), regular smoking status (OR = 0.62, 95%CI 0.44–0.88), obesity (OR = 0.70, 95%CI 0.52–0.94), lack of opportunity to practice PA (OR = 0.57, 95%CI 0.44–0.75), and lack of conditions to practice PA (OR = 0.73, 95%CI 0.57–0.94) were factors that contributed to poor adherence to PA. In contrast, a university education (OR = 2.67, 95%CI 1.70–4.19), the highest category of income (OR = 1.96, 95%CI 1.50–2.57), and regular alcohol use (OR = 1.99, 95%CI 1.05–3.76) were factors that positively contributed to adherence to PA in men. After adjustment for all of those factors, similar results were found except for years of education, alcohol use, and conditions to practice PA in the neighborhood. In the crude analyses, years of education, income, alcohol use, BMI, and opportunity and conditions to engage in PA led to the same behavior in women with hypertension as in men with hypertension. However, individuals with poor or very poor self-perceived health had lower adherence. Other factors that contributed to poor adherence to PA in hypertensive women included the presence of three risk factors (OR = 0.59, 95%CI 0.36–0.96), the presence of joint disease (OR = 0.71, 95%CI 0.56–0.92), and the lack of seeing other persons exercising (OR = 0.61, 95%CI 0.44–0.83). After adjusting for all of the factors previously mentioned, we observed a significant negative association between adherence to PA and only poor self-perceived health. In the opposite direction, we observed significant positive associations of adherence to PA in hypertensive women with high educational level and income.

Among those with diabetes, having a university education and income above USD 505.00 increased the odds of adherence to the PA recommended levels, and having a fair self-perceived health status lowered these odds. In the stratified analysis by gender, men who had the no opportunity to practice PA were 49 times less likely to be adherent to PA than those with the opportunity; additionally, diabetic women who neither agreed nor disagreed to seeing persons practicing PA were three times more likely to adhere to PA than diabetic women who agreed. In the multivariable models, only high income had a significant association with adherence to PA in women. Overall, high income and high education level contributed positively to adherence to PA, while a fair self-perceived health status contributed negatively.

## DISCUSSION

Despite the importance of regular PA for the prevention and treatment of many chronic diseases, few studies have investigated the factors that affect adherence to PA in subjects with these diseases^15^. Notably, these factors may vary across populations, and such differences should be taken into account by health providers and professionals when planning and supervising exercise activities. The results of this study showed that there is low (less than 20%) adherence to PA recommendations in adults with a previous diagnosis of dyslipidemia, hypertension, or diabetes. Similar results have been found by Fang et al.^16^ (43.1% *versus* 51.7%) and Churilla et al.^7^ (59.1% *versus* 68.3%), who have reported that dyslipidemic adults were less likely to regularly engage in PA than those without dyslipidemia. Hays and Clark^17^ have also observed a low prevalence of PA in adults with type 2 diabetes.

The WHO has promoted regular and long-term PA as an intervention that can reduce the global rates of morbidity and mortality from cardiovascular diseases. Accordingly, the Global Action Plan for the Prevention and Control of NCD 2013–2020 aims to achieve a 10% reduction in the rates of physical inactivity^18^. It is important to note that the *ELSA-Brasil* cohort study is not representative of the Brazilian population, as the participants had a higher education level and income than the general population. Despite these characteristics, our data showed that only a small proportion of the subjects of the study regularly participated in PA programs. A better understanding of the variables explaining this low adherence may help health providers in the development of strategies to encourage specific populations to improve their adherence to exercises, especially considering the large and growing body of evidence demonstrating the numerous health benefits of regular PA. Physical activity is associated with a reduced risk of all-cause mortality in hypertensive adults and a reduced incidence of cardiovascular events, microvascular complications, and all-cause mortality in diabetic patients^19^ ^,^ ^20^. Madden et al.^21^ have shown that relatively short aerobic exercise interventions in older adults reduced the arterial stiffening associated with aging, diabetes, and hypercholesterolemia and they should thus be included as first-line treatment for these conditions. Additionally, PA is a key determinant of energy expenditure and therefore plays a fundamental role in weight control^22–24^. Considering the high prevalence of overweight and obesity in Brazil and the world^25^, it is important to increase the number of individuals who regularly attend PA programs to reduce the increasing incidence of diabetes and other weight-related chronic diseases.

A number of different factors can interact to create barriers to adequate exercise. In our study, adherence to PA recommendations was lower for those aged 35 to 64 years, although it was greater in men aged 65–74 years. However, a recent study with men and women aged 70–93 years from 25 towns in the United Kingdom has found that few participants reached the current PA guidelines. Those who adhered to the guidelines were younger, had fewer chronic health conditions, and had less severe mobility limitations^26^. We also observed that decreased mobility from joint diseases was a limiting factor for adherence to PA in dyslipidemic women.

The male gender, a higher socioeconomic condition, and a higher education level have been reported to increase LTPA in Brazilian individuals^27^. Our data confirmed this finding and demonstrated that women were less active than men regardless of health condition. Another study showed that men were more likely to be engaged in PA at guideline-recommended levels^8^. Regarding socioeconomic condition, our results showed that the adherence to PA was higher in individuals with university education and in those in the highest income group. In the fully adjusted model, education and high income were the only factors that favored adherence to PA in dyslipidemic, hypertensive, and diabetic individuals. In support of our results, Dontje et al.^28^ have shown in a longitudinal study of middle-aged women with chronic diseases that the odds of being inactive were lower among those with a higher educational level. The gender differences found in our study are worth noting because they may suggest the need to adopt different strategies to improve adherence to PA in men and women; this type of adaptation is important when considering public policies for the whole population. One factor that limited adherence to PA was the self-perception of fair or poor health. Given the cross-sectional nature of our study, a causal relationship between these two variables could not be established. However, the perception of poor health may represent a robust negative psychological influence on exercise engagement. The fear of worsening previous diseases may be stronger than the appeal of attaining the well-known benefits of exercise on health status. Conversely, we observed that individuals who self-reported hypertension, diabetes, and dyslipidemia were more likely to engage in LTPA when they had better self-perceived health status. Alkerwi et al.^29^ have shown that, in three European regions, the awareness of the positive health effects of PA may be crucial to motivating persons to become more active. Therefore, the benefits of regular exercise for chronic diseases must be better explained to those with and without a positive self-perception of health.

The BMI also affected adherence to PA in all three groups investigated. We found an inverse relationship between PA and BMI in men and women. Again, the subgroup that would receive the greatest benefit from exercise (i.e., those with higher BMI) showed lower adherence to PA. Healthy adults have been reported to perform more PA compared to unhealthy obese adults^30^. The odds of inactivity were higher for women with a higher BMI, lower for former smokers, and higher for current smokers^28^. Our study also indicated that regular smoking could be associated with low adherence to PA recommendations. The results of Churilla et al.^7^ have also shown that the prevalence of inactivity was greater among current smokers than in former or never smokers.

Participants with a negative self-perception of their conditions and those who did not have the opportunity to exercise in their neighborhood had lower adherence to PA. Therefore, our data support the importance of improving urban mobility and the provision of adequate facilities to help persons practice PA close to where they live, especially for priority groups, such as older adults, individuals who are obese, and individuals who have diabetes, hypertension, and other chronic diseases.

In summary, the proportion of adults with diabetes, dyslipidemia, and hypertension who were adherent to the PA recommendations remained low. Only one out of five received the benefits that PA could have for their health. Men were more active than women when these diseases were present. The main conditions that favored adherence to PA were higher levels of income and education. Subjects with a negative self-perception of their health and those who did not have the opportunity to exercise in their neighborhood also showed lower adherence to PA. Older age, excessive body weight, fair self-perceived health, and regular smoking were also associated with decreased adherence. These findings are crucial for helping decision makers implement effective prevention strategies, target high-risk groups, and control and treat chronic diseases.

### Study Limitations

Our study has some limitations. Physical activity was evaluated by a questionnaire rather than by objective measurements. Moreover, PA in this study included only exercise performed during leisure time. Therefore, it did not include all energy expenditures, which could be from active transportation (e.g., walking, biking), household work, or occupational work. Moreover, the cross-sectional nature of the data allowed associations but not causal inferences. Finally, we used self-report to determine dyslipidemia, hypertension, and diabetes because the main objective of the study was to evaluate adherence to PA in individuals aware of these chronic conditions. However, this strategy may have excluded a substantial number of undiagnosed subjects.
